# Implications of SARS-COV-2 infection in the diagnosis and management of the pediatric gastrointestinal disease

**DOI:** 10.1186/s13052-021-01020-9

**Published:** 2021-03-24

**Authors:** Valeria Dipasquale, Stefano Passanisi, Ugo Cucinotta, Antonio Cascio, Claudio Romano

**Affiliations:** 1 Pediatric Gastroenterology and Cystic Fibrosis Unit, Department of Human Pathology in Adulthood and Childhood “G. Barresi”, University of Messina, Via Consolare Valeria, 98124 Messina, Italy; 2 Department of Health Promotion, Mother and Child Care, Internal Medicine and Medical Specialties, University of Palermo, Palermo, Italy

**Keywords:** ACE2, COVID-19, Digestive endoscopy, Functional gastrointestinal disorders, Gastrointestinal symptoms, Inflammatory bowel disease, Liver disease, Pediatrics

## Abstract

Gastrointestinal diseases such as celiac disease, functional gastrointestinal disorders (FGIDs), inflammatory bowel disease (IBDs) and acute or chronic diarrhea are quite frequent in the pediatric population. The approach, the diagnosis and management can be changed in the 2019 coronavirus disease (COVID-19) pandemic era. This review has focused on: i) the current understanding of digestive involvement in severe acute respiratory syndrome coronavirus 2 (SARS-CoV-2) infected children and adolescents and the clinical implications of COVID-19 for pediatric gastroenterologists, ii) the impact of COVID-19 on the clinical approach to patients with pre-existing or onset diseases, including diagnosis and treatment, and iii) the role and limited access to the instrumental diagnosis such as digestive endoscopy. To date, it is unclear if immunosuppression in patients with IBD and chronic liver disease represents a risk factor for adverse outcomes. Scheduled outpatient follow-up visits may be postponed, especially in patients in remission. Conversely, telemedicine services are strongly recommended. The introduction of new therapeutic regimens should be made on an individual basis, discussing the benefits and risks with each patient. Furthermore, psychological care in all children with chronic disease and their parents should be ensured. All non-urgent and elective endoscopic procedures may be postponed as they must be considered at high risk of viral transmission. Finally, until SARS-CoV-2 vaccination is not available, strict adherence to standard social distancing protocols and the use of personal protective equipment should continue to be recommended.

## Introduction

The 2019 coronavirus disease (COVID-19), caused by severe acute respiratory syndrome coronavirus 2 (SARS-CoV-2), has created unprecedented global challenges from public health, economic, medical, and research standpoints. As of December 8, 2020, more than 68,000,000 cases and more than 1,550,000 deaths all over the world have been reported (https://www.worldometers.info/coronavirus/). Italy, as well as most of the European countries, is currently overwhelmed with a second pandemic wave that is characterized by a drastic reduction in the median age of infected subjects compared to the first pandemic wave occurring from March to May, 2020. The rate of pediatric patients diagnosed with SARS-CoV-2 is also much greater than the early stages of the pandemic (11.7% versus 1.5%) (https://www.epicentro.iss.it/coronavirus/). These data are likely related to an overt policy of population-wide testing paired with contact tracing [1]. Fortunately, children with COVID-19 seem to have milder disease course in comparison to adults [2]. Many pediatric COVID-19 patients have nasal obstruction, fever, runny nose, pharyngalgia, whereas signs of respiratory failure rarely occur [3]. Conversely, gastrointestinal symptoms have been demonstrated to be relatively common in pediatric COVID-19 patients [4]. Here we present a summary of: i) the current understanding of the gastrointestinal involvement in SARS-CoV-2 infected children and adolescents, and ii) the impact of COVID-19 on the clinical approach to patients with gastrointestinal disease, including diagnosis and treatment, and iii) the role and limited access to the instrumental diagnosis such as digestive endoscopy, in order to provide an overview of the clinical implications of COVID-19 for the pediatric gastroenterologist.

## Gastrointestinal symptoms in children with SARS-CoV-2 infection

Since the early stages of the SARS-CoV-2 outbreak, the involvement of digestive tract has been documented. A recent systematic review showed that digestive symptoms were present in 17.7% of a total of 3028 infected pediatric patients. Vomiting, nausea, diarrhea, and anorexia were the most common gastrointestinal symptoms [4]. Another very recent systematic review with meta-analysis reported an overall prevalence of anorexia of 18%, followed by diarrhea (15%), nausea and vomiting (10%), and abdominal pain (6%) [5]. It is noteworthy that the gastrointestinal expression of SARS-CoV-2 infection is non-specific and, even associated with fever, can mimic infections common in childhood, such as acute gastroenteritis. This led clinicians to underestimate (and under-report) these symptoms, mainly during the first pandemic wave, and it could affect the overall incidence of digestive symptoms in pediatric COVID-19 patients.

The intestinal tropism of SARS-CoV-2 was suspected after the identification of viral RNA in a stool specimen in the first case described in the United States [6]. The main determinant of SARS-CoV-2 infection is considered to be the spike glycoprotein, which binds to the extracellular portion of angiotensin-converting enzyme 2 (ACE2) on host cells [7, 8]. High expression levels of ACE2 have been demonstrated in the brush border membrane of small intestine enterocytes, especially in proximal and distal enterocytes [9]. Moreover, the presence of cellular serine proteases, transmembrane protease serine 2 (TMPRSS2) and transmembrane protease serine 4 (TMPRSS4) promotes SARS-CoV-2 infection of enterocytes by cleaving the spike glycoprotein of the virus on the cell membrane [10]. ACE2 has a central role in regulation of intestinal amino acid homeostasis, innate immunity, gut microbial ecology, and transmissible susceptibility to colitis [11]. Therefore, mutual interaction between SARS-CoV-2 and ACE2 might disrupt the function of ACE2 and result in inflammation and diarrhea.

Regarding nausea and vomiting, it has been proposed that emetic mechanisms might be activated by mediators released from the intestinal epithelium by SARS-CoV-2, which modulate vagal afferents and activate the area postrema. Both the vagal afferents and area postrema efferents activate the nucleus tractus solitarius, which in turn activates the visceral and somatic motor pathways for vomiting and also sends projections to higher brain regions leading to the appearance of nausea [12].

In terms of prognosis, gastrointestinal symptoms are increasingly recognized to be associated with the presentation of pediatric multisystemic inflammatory syndrome, resembling Kawasaki disease, which is a life-threatening condition. A multicenter, descriptive, observational study including 101 pediatric COVID-19 patients demonstrated that gastrointestinal symptoms were predictive of severity in COVID-19 children admitted to hospitals [13]. However, further prognostic studies are awaited.

In children with diarrhea, abdominal pain, nausea, vomiting, and other gastrointestinal symptoms, attention should be paid to their epidemiological history, with screening of suspected patients. In daily life, good hygiene practices, such as washing hands frequently and closing the toilet lid when flushing, should be implemented.

## SARS-CoV-2 and gastrointestinal diseases

### SARS-CoV-2 and inflammatory bowel disease

It is well known that patients with inflammatory bowel disease (IBD) have an increased risk of infections due to multifactorial immunological impairment [14]. Furthermore, ACE2 is particularly highly expressed in intestinal epithelial cells from the terminal ileum and to a lesser extent in the colon, where mucosal inflammation in patients with IBD is frequently detected [15]. Plasma ACE2 activity is also higher in patients with IBD compared to healthy controls, with a trend towards higher levels in patients with Crohn’s disease than ulcerative colitis [16]. Finally, pro-inflammatory cytokines expressed in IBD may further increase the expression of ACE2 [17] that is the entry receptor for SARS-CoV-2, as above discussed. Based on these findings, patients with IBD might be considered highly susceptible to COVID-19. Conversely, real-life experiences demonstrated that IBD patients seem to be less susceptible to SARS-CoV-2 infection. A recent study on 1918 adult IBD patients showed that cumulative incidence of COVID-19 was 6.1 per 1000 subjects, demonstrating that IBD patients were less likely to be infected than the general population, with an odds ratio of 0.74 (95% CI 0.70–0.77; *P* < 0.001) [18]. Another analysis from the Surveillance Epidemiology of Coronavirus Under Research Exclusion for Inflammatory Bowel Disease (SECURE-IBD) on 209 pediatric IBD patients infected by SARS-CoV-2 showed that there were no deaths in the study population, and only 14 children (7%) were hospitalized, of whom 2 (1%) required mechanical ventilation [19]. Similarly, preliminary data (June 2020) from the Porto and Interest-group of ESPGHAN, showed that eight IBD children had COVID-19 globally, all with mild infection without needing hospitalization despite treatment with immunomodulators and/or biologics [20]. Although it has been proposed that the lower infection rate may be a consequence of improved adherence with shielding recommendations [18], the reasons why IBD patients appear to be less affected and if infected develop milder clinical pictures are still unknown.

Some key points have been addressed to better guide clinical gastroenterologists in the care of patients with IBD. In this context, the use of corticosteroids has been widely debated. Although steroids are currently being used to treat patients with moderate-to-severe COVID-19 in order to counteract the super inflammation state in the later phases of the disease, their use may increase viral burden in the early stages of the infection [21]. This hypothesis has been confirmed by a recent analysis including 525 patients with IBD, which revealed that steroid therapy was the most significant factor for the development of severe COVID-19 [22]. While the use of corticosteroids remains recommended as induction therapy in IBD children, steroid weaning should be made on an individual basis, discussing the benefits and risks with each patient, and it should be conducted under strict medical surveillance [23]. Exclusive enteral nutrition has showed to be as effective as steroid therapy in inducing remission in CD pediatric patients, both in the first flare-up and during relapses of symptoms [24]. Thiopurines (azathioprine or 6-mercaptopurine) are recommended as one option for maintenance of steroid free remission in children at risk for poor disease outcome [24]. Data from the SECURE-IBD reported that compared with TNFα antagonist monotherapy, thiopurine monotherapy and combination therapy with TNFα antagonist and thiopurine were associated with an increased risk of severe COVID-19 [25]. Another object of concern for physicians has been the use of biologics due to possible infectious risks related to immunosuppression. In particular, antibodies targeting TNFα may negatively influence antiviral immunity. However, the use of anti-TNFα has not been associated with adverse outcomes in patients with IBD thus far [22]. During the first pandemic wave, it has been reported that 21–23% of pediatric patients who delayed or temporarily discontinued biologics therapy due to the lock-down period, experienced a disease exacerbation [26]. Therefore the use of biologics should be started and/or regularly continued, as well as there is no evidence to consider discontinuing ongoing therapy or not recommending the beginning of conventional immunomodulators [21].

Telemedicine services are strongly recommended, particularly for those IBD patients with stable disease on maintenance therapy in order to minimize hospitalizations, outpatient gastroenterology visits and, consequently, SARS-CoV-2 exposure risk [27, 28].

### SARS-CoV-2 and chronic liver diseases

Both hepatocytes and cholangiocytes express ACE2 receptors, thereby making the liver a potential target for SARS-CoV-2 infection [29]. Liver involvement in infected patients has been described, especially in severe COVID-19 cases [30]. Liver disease manifests mainly with elevated aminotransferase levels and slightly elevated bilirubin levels [29]. However, liver function test disorders are rarely described in infected children [31]. Regarding pediatric patients with preexisting liver disease, it is still not clear whether these subjects are more susceptible to SARS-CoV-2 infection. Although patients with advanced liver disease may be considered at increased risk of infection due to the use of immunosuppressive therapy, corticosteroids, and cirrhosis-induced immunodeficiency, real-life experience data did not show a more severe clinical course of COVID-19 and increased mortality among these children [32]. Therefore, it has been hypothesized that immunosuppression could be protective against SARS-CoV-2 infection and its complications, mainly driven by a well-documented pro-inflammatory state. A recent report on a child suffering from autoimmune hepatitis and type 1 diabetes with COVID-19 showed that low doses of prednisolone might suppress activated regulatory T cells, regulatory B cells, and IL-6 production and therefore permitting the activation of CD8 T cells, eliminating the virus [33].

Children with chronic liver disease (e.g. autoimmune liver disease, hepatitis C, liver transplanted patients) must strictly adhere to standard isolation protocols to avoid coming into contact with SARS-CoV-2. Seasonal flu vaccination may be strongly recommended (as well as in IBD pediatric patients) [34]. In patients with stable disease, follow-up visits should be postponed. Conversely, web-based and telephone-based consultations may be used. Routine laboratory investigations may be performed in a local laboratory, while liver-diagnostic procedures should be avoided unless required by an emergency condition such as severe flares of disease, obstructive jaundice, and gastrointestinal bleeding [34, 35]. The immunosuppressive therapy should not be reduced or stopped to avoid acute flares of disease and unnecessary admission to the hospital [36]. In patients with chronic hepatitis C already on treatment with oral direct-acting antiviral, therapy should be continued [37]. On the other hand, the introduction of a direct-acting antiviral regimen should be based on individual cases. While in patients with stable chronic hepatitis C therapy should be postponed after the pandemic wave, in selected cases with a clinically documented advanced liver disease it is reasonable to start direct-acting antiviral treatment [35]. To ensure therapy maintenance, organizing drug dispensation with a local pharmacy or home delivery may be recommended.

### SARS-CoV-2 and functional gastrointestinal disorders

Based on the Rome IV criteria, functional gastrointestinal disorders (FGIDs) are largely present in the pediatric population. Particularly, 24.7% of infants and toddlers aged 0–3 years and 25.0% of children and adolescents fulfill symptom-based criteria for FIGDs diagnosis [38]. Functional constipation is the most common among these disorders, followed by abdominal pain not otherwise specified, and disorders of nausea and vomiting [39]. These manifestations overlap with gastrointestinal symptoms of SARS-CoV-2 infection. Therefore, pediatricians and emergency physicians should make every effort to pay attention to patients diagnosed with FGIDs presenting with flares of their symptoms. These patients should undergo a careful epidemiological investigation and, if available, a naso-pharyngeal test.

Gastrointestinal infections caused by viruses (e.g. Norwalk-like viruses and Rotavirus) have been reported to be related to the appearance of post-infectious irritable bowel syndrome (PI-IBS), as well as post-infectious functional dyspepsia [40, 41]. Some clinical peculiarities of gastrointestinal involvement of SARS-CoV-2 such as long duration of diarrhea, weight loss, antibiotics therapy, along with psychological distress, seem to carry to a higher risk for PI-IBS [42, 43]. The impact of SARS-CoV-2 infection on the occurrence of PI-IBS should be carefully evaluated in the near future. Thus, pediatric patients presenting with chronic abdominal pain associated with a change in the frequency or form of stool should undergo SARS-CoV-2 serological test, particularly those who have suffered from gastrointestinal disorders in recent months.

### Psychological functioning

An underestimated aspect of the COVID-19 pandemic has been the psychological impact on the population. Lock-down forced people to radically change their daily lifestyles, exposing them to a high risk of developing feelings of panic, anxiety, and depression. Children and adolescents experienced behavioral and emotional disorders due to the strong experience of physical and social isolation [44]. Psychological issues have been mostly found among subjects with preexisting chronic diseases [45, 46]. Therefore, psychological assistance in all children with IBD and their parents with the aid of online video-psychotherapy sessions should be ensured [21].

The biopsychosocial model, a framework that integrates the biological and psychosocial processes, is currently the most accepted paradigm to clarify the pathogenesis of FGIDs. According to this pathogenetic model, psychological distress may play a crucial role in the development of FGIDs [47]. As discussed above, the COVID-19 pandemic is widely considered a stressful life condition for children and adolescents. Strict measures to counteract the community-based viral spread have been a further burden for physical and mental health. A recent Japanese study demonstrated how COVID-19 pandemic negatively affected patients with functional dyspepsia and irritable bowel syndrome. It was reported that 11.9% of subjects reported deterioration of gastrointestinal symptoms, which was significantly associated with psychological disease comorbidity, and stress at work/school [48]. Conversely, in another study conducted on 71 pediatric patients with celiac disease, the prevalence of FGIDs has been reported to decrease during the COVID-19 lockdown. The authors explained these findings by the assumption that the occurrence of gastrointestinal symptoms in these subjects could be influenced by some psychosocial aspects, such as an increased parental closeness [49]. Anxiety and depression are also considered risk factors for the development of PI-IBS [42].

In summary, COVID-19 had a variable impact on the diagnosis and clinical management of patients with gastrointestinal diseases (Table 1).

**Table 1:** The management of pediatric gastrointestinal diseases in the era of SARS-CoV-2 pandemic

Pediatric gastrointestinal diseases in the era of SARS-CoV-2	
• Corticosteroids may be used as induction therapy in IBD children, and steroid weaning should be made on an individual basis	
• Treatment with biologics or other conventional immunomodulators should be started and/or regularly continued in IBD pediatric patients	
• Patients with autoimmune hepatitis should not reduce or stop the immunosuppressive therapy to avoid acute flares of disease and unnecessary hospital admission	
• In patients with chronic hepatitis C already on treatment with oral direct-acting antiviral, therapy should be continued	
• Children and adolescents presenting with chronic diarrhea and abdominal pain and a recent history of gastrointestinal disorders should undergo serological SARS-CoV-2 testing	
• Antitransglutaminase IgA threshold for biopsy-sparing approach for diagnosis of celiac disease may be reduced to ≥5 times upper limit normal	
• Psychological assistance services for patients suffering from chronic gastrointestinal diseases and their parents should be ensured	

*IBD* inflammatory bowel disease, *SARS-CoV-2* severe acute respiratory syndrome coronavirus 2.

Children and adolescents suffering from gastrointestinal diseases were often unable to comply with scheduled outpatient follow-up visits due to strict social distancing and self-isolation measures. Telemedicine services have been strengthened to ensure clinical assistance for patients and their parents. Most diagnostic procedures were postponed unless required by an emergency condition.

## Digestive endoscopy

All gastrointestinal endoscopic procedures must be considered at high viral transmission risk. In particular, upper digestive tract endoscopy can lead to airborne transmission by causing coughing, gagging and retching, while passing flatus and pathogen-carrying liquid stools can occur during colonoscopy [50]. Although it is accepted that all non-urgent and elective procedures may be postponed, the limitation of endoscopic services is related to possible negative outcomes such as delayed diagnoses and psychological distress [51]. The 2020 European Society of Pediatric Gastroenterology Hepatology and Nutrition guidelines for celiac disease have allowed a biopsy-free approach in endomysial antibodies positive children with high serum antitransglutaminase (TGA)-IgA titer (> 10 time upper limit of normal), being esophagogastroduodenoscopy still necessary for diagnosis in children with lower title [52]. However, it has been suggested a temporary reduction of the IgA threshold (≥ 5 times upper limit normal) for biopsy-sparing approach for diagnosis of celiac disease during the COVID-19 pandemic [53]. Multiple national and international gastroenterology and endoscopic scientific societies agree with the need to perform endoscopy in the following urgent cases: acute gastrointestinal bleeding, upper gastrointestinal foreign bodies requiring removal, nutrient support (application of percutaneous endoscopic gastrostomy/jejunostomy), obstructing upper or lower gastrointestinal lesion that requires stenting/therapy. Regarding IBD patients, endoscopic procedures should not be deferred in cases of confirmation of new diagnoses, severe acute flares, or occurrence of complications [21, 54–56]. If available, negative pressure rooms should be used, especially for patients infected by SARS-CoV-2 [57, 58]. In all cases, assessment and screening for signs of infections, travel history, contact with potentially infected patients must be investigated at hospital admission. Patients should undergo a naso-pharyngeal swab test 24–48 h before endoscopic procedures which are performed under deep sedation and/or general anesthesia, as usually occurs in children [21]. During the procedure, all endoscopy staff members must wear personal protective equipment and frequently wash their hands with alcoholic solutions before and after procedures. Previous and successive cleaning of the equipment and of the operating room should always be ensured [54, 55]. Current recommendations are summarized in Table 2.

**Table 2:** Performing digestive endoscopy during the COVID-19 pandemic

Digestive endoscopy in the SARS-CoV-2 era	
• Endoscopy must be performed in all urgent cases; elective procedures should be deferred	
• Endoscopic procedures should not be postponed in cases of new diagnoses of inflammatory bowel disease and the occurrence of severe acute flares or complications	
• All patients should be tested by naso-pharyngeal swab 24–48 h before endoscopic procedures	
• Negative pressure rooms should be used for with confirmed SARS-CoV-2 infection	
• All endoscopy staff members must wear personal protective equipment and frequently wash their hands with alcoholic solutions before and after procedures	
• Endoscopic room and equipment should be sterilized after each patient’s procedure	

*COVID-19* coronavirus disease 2019, *SARS-CoV-2* severe acute respiratory syndrome coronavirus 2.

Even after the second pandemic wave, strict adherence to universal precautions and use of personal protective equipment should be maintained, especially in areas with high rates of SARS-CoV-2 transmission.

## Conclusions

COVID-19 pandemic has had an important impact on the practice of medicine and the approach to patient care worldwide. The role of pediatric gastroenterologist is linked not only to the identification of digestive symptoms suggestive of SARS-CoV-2 infection, but also to the evaluation of the most suitable management plan for children and adolescents with pre-existing chronic gastrointestinal diseases. Some trials are ongoing to assess the safety of and immune response to the COVID-19 vaccine in children and adolescents aged 6–17 years (AstraZeneca) and 12 years and older (Pzifer, Moderna). Until vaccination against SARS-CoV-2 is not available, the development of standardized management protocols for chronic diseases is needed, along with patient education recommendations on risk and precaution of COVID-19.

## Data Availability

Unpublished experimental data are not included in this review.

## Abbreviations

ACE2: angiotensin-converting enzyme 2
COVID-19: 2019 coronavirus disease
FGIDs: functional gastrointestinal disorders
IBD: inflammatory bowel disease
PI-IBS: post infectious irritable bowel syndrome
SARS-CoV-2: severe acute respiratory syndrome coronavirus 2
TMPRSS2: transmembrane protease serine 2
TMPRSS4: transmembrane protease serine 4
